# Endometriosis as a highly relevant yet neglected gynecologic condition in Asian women

**DOI:** 10.1530/EC-23-0169

**Published:** 2023-10-05

**Authors:** Michael C Velarde, Mikaela Erlinda M Bucu, Maria Antonia E Habana

**Affiliations:** 1 Institute of Biology, College of Science, University of the Philippines Diliman, Quezon, Metro Manila, Philippines; 2 Department of Obstetrics and Gynecology, College of Medicine, University of the Philippines Manila, Metro Manila, Philippines

**Keywords:** endometrioma, endocrine-disrupting chemicals, food consumption, ethnic disparities

## Abstract

Endometriosis is a chronic, debilitating disease characterized by the growth of endometrial tissues outside the endometrium. Its prevalence seems to differ across ethnicities, with the disease affecting and presenting with advanced stages in Asians more than any other race. Despite this, data on endometriosis in Asians is limited, and there seems to be a lack of support for endometriosis research in Asia. Hence, this review aims to consolidate the available literature on endometriosis in Asians to identify the gaps in knowledge regarding its occurrence in this population and emphasize the need to address the disease in this part of the world. Certain genetic, dietary, and environmental factors that predominate in Asians compared to other ethnicities may potentially impact endometriosis. Understanding these differences is essential in providing innovative strategies for reducing health disparities in endometriosis incidence and presentation across ethnic groups, thus improving disease management and health outcomes.

## Introduction

Endometriosis is a debilitating, chronic, inflammatory, progressive, estrogen-dependent disease characterized by the presence of endometrial tissues outside the uterine cavity, presenting clinically as dysmenorrhea, dyspareunia, and dyschezia (1, 2). It has also been associated with an increased risk of asthma, autoimmune disorders, cardiovascular disease, and certain cancers (3). It is histologically characterized by the presence of functioning endometrial glands and stroma in extra-uterine sites, often resulting in a chronic, inflammatory reaction. The long-standing inflammation associated with the disease can cause extensive scarring, resulting in the distortion of the normal pelvic anatomy, which may cause severe life-impacting chronic pelvic pain during periods, sexual intercourse, bowel movements and/or urination, abdominal bloating, nausea, and fatigue, as well as depression, anxiety, and infertility (4). The chronic painful nature of pelvic endometriosis impacts various aspects of a woman’s life, including her school, work, social, and sexual and intimate relationships, leading to an overall decrease in quality of life (5, 6, 7, 8).

The delay in the diagnosis of endometriosis may take about a decade, as a definitive diagnosis is often established through surgery (9). Hence, affected women may live with the disease all throughout their reproductive years (10). Moreover, while several medications are available to manage the symptoms of endometriosis, laparoscopic excision surgery remains an important form of treatment, especially for improving fertility in women with endometriosis (4, 11). The difficulty in detection and the high cost of medications and treatments then become a strong financial burden to a woman with endometriosis. It is estimated that an affected woman would incur about an average of 9579 euros per year for the management of the disease (1). This further increases if the woman would miss work due to severe pain, as women with endometriosis tend to lose an average of 10.8 h of work per week due to the disease (12). Hence, the burden of the disease in women with endometriosis is likely heightened by their socioeconomic status, especially in low-income countries.

In Western populations, the prevalence of endometriosis can reach up to 10% of all reproductive-aged women, but estimates worldwide vary considerably, ranging from 2% to 45%, depending on the diagnostic criteria applied and the population examined (13). For example, the prevalence rates for endometriosis in fertile and infertile women are 0.5–5% and 25–40%, respectively (11). Low estimates may be due to studies that restrict inclusion criteria to patients with surgically confirmed endometriosis, and high prevalence estimates may be due to reports that include symptomatic women who were managed medically without tissue confirmation. While most studies do not report the type and stage of endometriosis, several studies describe a significantly higher overall prevalence of endometriosis in Asians than in other ethnicities (14, 15, 16), with estimates ranging from 6.8% to 15.7% (2, 17). Compared with Caucasian women, Asian women are more likely to be diagnosed with endometriosis (odds ratio (OR) 1.63, 95% CI 1.03–2.58) (14). Filipinos, Indians, Japanese, and Koreans are among the top Asian ethnicities who are more likely to have endometriosis than Caucasian women (17). In the annual census of the Department of Obstetrics and Gynecology of the Philippine General Hospital, endometriosis consistently comprises 15–20% of consults annually and 7.9% of surgeries for benign gynecologic diseases confirmed by histopathology. Despite the high rates of endometriosis in Asian women, data on endometriosis and funding support for endometriosis research in the region are still very limited.

## Clinical presentation and severity of endometriosis in Asian women

Women with endometriosis often present with dysmenorrhea, severe chronic pelvic pain, and infertility. Other symptoms include dyspareunia, as well as extragenital symptoms such as dyschezia, dysuria, hematuria, and rectal bleeding (18). While East and Southeast Asian women with endometriosis have lesser pain and better quality of life than Caucasians (19, 20, 21), they are more likely to present with ovarian endometriomas and deep infiltrating lesions, or moderate-to-severe endometriosis at the time of surgery. Indeed, one study noted a 10.9 times higher rate of moderate-to-severe cases of endometriosis with revised American Fertility Society (rAFS) scores of III–IV at the time of surgery (adjusted odds ratio (aOR): 10.87, 95% CI: 4.34–27.21, *P* < 0.001, *n* = 368) and a 4.1 times higher presence of ovarian endometriomas on ultrasound (aOR: 4.10, 95% CI: 2.68–6.26, *P* < 0.001, *n* = 1521) among East and Southeast Asian women compared with their Caucasian counterparts after adjustments for age, BMI, infertility, previous surgery for endometriosis and ever usage of hormonal medications (22). This is speculated to be due in part to their poor health-seeking practices, as East and Southeast Asian women in their study experience less pain than Caucasians and tend to be diagnosed at an older age, allowing lesions to progress over time and develop into a more severe form of the disease. It is important to point out, however, that there are still many factors to consider when comparing incidences and clinical presentations across ethnic groups, as it is still unclear whether these associations are mere artifacts of diagnostic biases or actual heterogeneity in endometriosis phenotype. Hence, further research is still warranted to confirm ethnic disparities in endometriosis.

Progestins and gonadotropin-releasing hormone (GnRH) analogs are some of the most used medications for suppressing the growth of endometriotic tissues (23). Intake of oral progestins, such as dienogest, inhibits estrogen-induced mitosis and shrinks endometriotic lesions, while continuous administration of GnRH agonists downregulates GnRH receptors and prevents the release of follicle-stimulating hormone and luteinizing hormone from the pituitary gland, resulting in a reduced estradiol production from the ovaries. However, there may be ethnic disparities in terms of response to medication and predisposition to treatment side effects in Asian vs Caucasian women. For example, while dienogest and GnRH analogs have similar efficacy in reducing endometriosis in Japanese and European women, dienogest decreases the total bone mineral density in Japanese but not European women, and GnRH analogs significantly reduced bone mineral density in European more than the Japanese cohorts (24). Whether this difference in medical side effects is also evident in other ethnic groups in Asia remains uncertain.

## Genetic polymorphisms in Asians implicated in endometriosis

The high concordance (75–87%) of the disease among identical twins suggests that genetic factors likely contribute to the development of endometriosis (25, 26). There are also reports showing a high incidence of endometriosis with certain family ties. Women with a first-degree relative diagnosed with endometriosis are highly likely to have the disease and highly likely to have severe endometriosis, possibly through a polygenic/multifactorial inheritance (27, 28, 29, 30). For example, in a larger cohort of women from Iceland, the familial clustering of endometriosis is evident even beyond first-degree relatives (31).

The key genes that contribute to the development of endometriosis remain elusive (32), but genetic polymorphisms of some genes have been described in women with endometriosis. One of the most studied genetic polymorphisms is that of estrogen receptors. In a study in Greece, women with endometriosis have a significantly higher frequency of a TC polymorphism recognized by the *Pvu*II restriction enzyme and a fewer median number of microsatellite repeat polymorphisms in the estrogen receptor alpha (*ESR1*) gene than women without the disease (33). The same association with the *Pvu*II polymorphism is observed in Taiwanese women (34) and Italian women (35). A meta-analysis suggests that this polymorphism in the *ESR1* gene may be associated with stage I–III endometriosis (36). However, other studies such as those done in China (37), Japan (38), and Germany (39) did not find an association between the *Pvu*II polymorphism of the *ESR1* gene and endometriosis. In contrast, polymorphisms in the estrogen receptor beta (*ESR2*) but not *ESR1* gene are associated with stage IV endometriosis in Japanese women (38). While the association of endometriosis with this polymorphism is found in women from Brazil (40), the link is not found in a group of Korean women (41). Hence, the contribution of these genetic polymorphisms to the pathogenesis of endometriosis remains controversial.

There are also other genetic polymorphisms that have been linked to a higher risk of endometriosis. An Arg399Gln mutation in the X-ray repair cross-complementing group 1 (XRCC1) is associated with endometriosis risk, with the A allele being a preventive factor for the disease in Asians but not among Middle Eastern women (42, 43). In addition, the pro-inflammatory cytokines TNF-α and interleukin-6 (IL-6) also contain polymorphisms that have been linked to endometriosis. The −1031T/C polymorphism in the TNF-α gene reduces the risk of endometriosis, while −238A/G and −174C/G gene polymorphisms in the TNF-α and IL-6, respectively, may increase the risk of endometriosis in Asians (44). However, these genetic polymorphisms were not detected in a more recent genome-wide association study of 60,674 European and East Asian women with endometriosis vs 701,926 controls (45). Instead, the extensive meta-analysis identified 42 genome-wide significant loci associated with endometriosis. Twelve of which were associated with pain sub-phenotypes (*P* < 0.05), including dysmenorrhea, dyspareunia, bladder pain, acyclical pelvic pain, and gastrointestinal pain. Two of the loci, namely 7p12.3/7p12.3 and VEZT/12q22, showed significant between-study heterogeneity due to ancestry after statistical adjustments (*P* < 0.05), with East Asian women having higher ORs for endometriosis on these two loci than European women, providing evidence that ethnicity can be a confounding factor for allelic effects on endometriosis. It is still unclear though if this is also true for endometriosis patients across different Asian countries. In addition, it is important to note that while the study described several genetic polymorphisms associated with endometriosis, a direct causal link between these mutations and the pathogenesis of endometriosis has not yet been demonstrated. More studies are then needed to show causal relationships.

## Asian diet implicated in endometriosis

Women with endometriosis tend to have a lower BMI than women without the disease (46). This association is even more pronounced in women with deep infiltrating endometriosis (47). Interestingly, women born with low birth weight (<2.5 kg or <5.5 lb) were also likely to be diagnosed with endometriosis (48). Because Asian women have lower BMI than Caucasians, the contribution of BMI to the high rates of endometriosis in Asians has been suggested. But while the reason for the paradoxical association between BMI and endometriosis remains unclear and its implication in the development of endometriosis in Asians is uncertain, it is well recognized that Asians have different dietary patterns than Caucasians. Given the major role of diet in controlling BMI, it is possible that diet may contribute to the disparity in the risk of endometriosis across ethnic groups.

Asians generally consume rice and noodles as the main sources of carbohydrates and tend to eat more nuts, legumes, and fishes than other ethnicities (49). Asians also eat more vegetables and strong spices as part of their diet. Likewise, Asians in the United States have the highest intake of rice and fishes among the various ethnic groups (50, 51). However, consumption of these food products has not been associated with an increased risk of endometriosis. In fact, several studies point to the potential beneficial effects of natural compounds found in these food groups (52, 53). For example, intake of long-chain omega-3 fatty acids, commonly found in fish, seaweeds, vegetable oils, and nuts, is linked to a lower likelihood of being diagnosed with endometriosis (54), given its ability to inhibit endometriotic endometrial cell survival and modulating cytokine expression (55, 56). *In vitro* and *in vivo* experiments suggest that exposure to phytoestrogens found in legumes and other plants reduces the risk of endometriosis due to their anti-estrogenic, antiproliferative, antiinflammatory, and proapoptotic effects (57). Moreover, a higher intake of green vegetables but lower consumption of beef, red meat, and ham is associated with a lower incidence of endometriosis (58). This may be in part due to the ability of high fiber to decrease the level of bioavailable estrogen, thus, lowering the risk of endometriosis (59). Hence, there may be other food components or lack thereof that contribute to the development of the disease.

Women with endometriosis have lower consumption of food rich in vitamins A, C, and E, zinc, and copper than women without the disease (60). Women with endometriosis also have lower total dairy food intake and reduced plasma 25(OH)D (vitamin D) levels than women without the disease (61, 62). While the administration of vitamin D has not yet been proven to be effective in treating endometriosis in women (63), it can reduce endometriosis development in mice, in part, by inhibiting endometrial cells to adhere to collagen and reducing macrophage recruitment and inflammatory cytokine secretion (64, 65, 66). Consistent with the higher incidence of endometriosis in Asians, several Asian populations, especially from South Asia, have average serum levels of 25(OH)D below 25 nM (67). Moreover, among the various ethnic groups in the United States, Asians consume the least amount of dairy in their diet (68). Since dairy products, especially milk, are often fortified with vitamin D and become an excellent source of the vitamin, Asians who do not consume much dairy often rely on other sources for the vitamin. Hence, it is possible that the low consumption of these nutrients may be an important factor for endometriosis in Asian women.

Despite the low BMI in Asians, Asians tend to have higher percent body fat than Caucasians at the same BMI (69, 70). This may be attributed in part to the consequence of consuming higher amounts of sodium, saturated fat, and cholesterol by Asians vs Western individuals (71). While there are no reports on whether Asian food with high carbohydrates, sodium, and cholesterol contributes to the progression of endometriosis, eating fatty foods, especially a diet with high trans-unsaturated fat, is associated with an increased risk of endometriosis (54, 72). This is consistent in an animal model of endometriosis wherein feeding mice with a high-fat diet results in more ectopic lesions (73). Women with endometriosis also tend to have higher levels of total cholesterol and mean low-density lipoprotein to total cholesterol ratios than women without the disease (74, 75). These high levels were more pronounced in women with moderate-to-severe endometriosis than in women with minimal-to-mild endometriosis (76, 77). Moreover, while BMI is inversely proportional to the risk of endometriosis, obesity does not protect from endometriosis (78). In fact, obese individuals with endometriosis have higher rAFS scores than endometriosis patients with lower BMI (79). Hence, while Asians have lower BMI than Caucasians, Asians may be consuming more fatty food which increases their risk of endometriosis. More studies are still needed to show the connection between the Asian diet and endometriosis.

## Environmental contaminants in Asia implicated in endometriosis

Many environmental contaminants, including heavy metals, are implicated in the pathophysiology of endometriosis. For example, high levels of lead are associated with an increased OR for endometriosis (80, 81), and co-exposure with cadmium further increases hospital admissions due to endometriosis (81). Notably, Asians have higher blood and urine concentrations of these heavy metals than other ethnicities living in the United States (51), with the high levels being more pronounced in Asians born outside the country (82). Hence, heavy metal exposure may be a potential contributor to endometriosis in Asians.

One possible reason for the disparity in heavy metal exposure in Asians vs other ethnicities may be due to their lifestyle differences. Rice, the staple food of most Asians, is a major source of inorganic arsenic (51, 82), while certain spices, particularly turmeric, used by many South Asians may be a potential source of lead exposure (83). Fish, an important protein source of many Asian cuisines, is the most common source of total arsenic exposure in Asians (82). Vegetables, cereal grains, and fruits may also be contaminated with cadmium and may be a potential source of exposure for Asian women (51, 82). Hence, while some of these foods are protective against endometriosis, heavy metal contamination in some of these products may counter their beneficial effects.

Endocrine-disrupting chemicals (EDCs), which are ubiquitous in the environment and found in many consumer products, have also been implicated in endometriosis (84). EDCs, such as bisphenols, phthalates, and per- and polyfluorinated substances (PFAS) can leach out from food packages and canned beverages and enter humans through oral ingestion. They then bind to hormone receptors to activate and inhibit receptor function or interact with membrane proteins to alter hormone synthesis and secretion (85). As endometriosis is an estrogen-dependent disease, certain EDCs that mimic estrogen may exacerbate the development and progression of endometriotic lesions. For example, bisphenol A (BPA) is higher in urine and serum of women with endometriosis (86, 87). Exposure to PFAS, a persistent organic pollutant, is associated with an increased risk of endometriosis in the United States (88, 89) and China (90). A meta-analysis further reveals an overall OR of 1.41 (95% CO: 1.23–1.60) for endometriosis across all exposures to BPA, polychlorinated biphenyls, organochlorine pesticides, and phthalate ester (91). In mice, the association between EDCs and endometriosis is also evident, showing that oral consumption of BPA increases the growth of ectopic endometrial lesions (92), and *in utero* exposure to BPA yields ovarian lesions reminiscent of endometriosis (93).

Data showing the relationship of EDCs with endometriosis in many Asian countries are still limited, but human exposure to several EDCs is very evident in this part of the world and has been detected since the late 1900s (94). While EDC exposure is a worldwide issue and not restricted to one country or ethnic group, the level of exposure to these contaminants may vary across the globe. For example, the daily intake and urinary concentrations of BPA in Western countries are generally higher than in many Asian countries (95, 96), but BPA exposure appears to be rising in Asia while gradually declining in Western countries (96). In fact, one study showed that while urinary BPA concentrations in Los Angeles residents decreased from 2012 to 2017, BPA levels remained the same in Beijing residents during this period (97). The same study further demonstrated that individuals who traveled from Los Angeles to Beijing during the study period had a 2.91-fold increase in their urinary BPA levels, which fully returned to baseline after going back to Los Angeles. In addition, there is a concerning trend of escalating PFAS levels in women from China (98, 99) and Korea (100, 101) and a persistent PFAS exposure documented in women from Vietnam and Japan (101, 102). In the Philippines, the level of BPA in Filipinos is about twice as much and the level of PFAS can reach up to ten times as high as in women from the United States (103). While the main sources of human exposure remain unclear, some studies suggest that manufacturing sites and food contamination may be important sources of human exposure to EDCs in Asia (104, 105).

The disparity in EDC exposure may be due in part to differences in local production, uses, and regulatory policies related to EDCs. For instance, there has been an increased production of EDC-containing goods in Asian countries like China, coincidental with the rise of EDC exposures in their population (99, 105). Moreover, as Western countries continue to implement effective restrictions on EDCs (106), some EDC-containing products may end up in many Asian countries, especially Southeast Asian countries with poor regulations (99, 107). Hence, it is imperative to have a concerted global effort to manage EDCs worldwide and to monitor the levels of EDCs and other emerging contaminants in each country, especially in vulnerable countries, to create relevant local policies and help lower the risk of diseases, such as endometriosis.

## Conclusion

**Figure 1:**
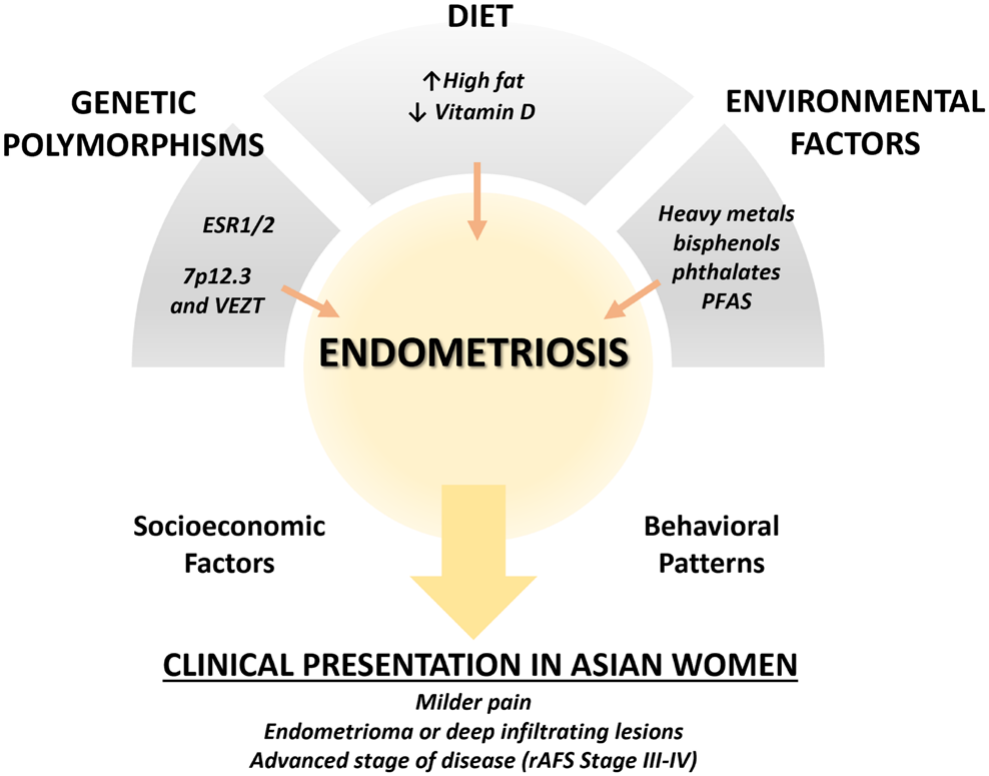
Endometriosis in Asian women. Genetic polymorphisms, dietary patterns, and environmental exposures that are characteristic of the region potentially influence the pathogenesis of endometriosis in Asian women. These elements, coupled with socioeconomic factors and behavioral patterns, result in a more advanced clinical presentation of the disease at the time of diagnosis. PFAS, per- and polyfluorinated substances.

The higher prevalence of endometriosis in Asians yet limited data on this population emphasizes the need for further investigation. Potential factors that predispose Asian women to a more advanced course of the disease should be explored further. Overall, the role of genetics, food consumption, and environment are important in influencing the pathogenesis of endometriosis in Asians (Fig. 1). Several modifiable factors that influence the development of endometriosis should then be explored in this region. Dietary therapy through a combination of vitamins, minerals, salts, lactic ferments, and fish oil may offer protective roles against the disease, as it seems to be as equally effective as hormonal therapy in reducing non-menstrual pelvic pain in endometriosis patients after surgery (108). Strategies to reduce environmental contaminants from important food sources, such as fish, would provide a universal benefit to society. Other emerging contaminants of concern, including microplastics (109), should also be investigated. Determining the impact of genetics, food consumption, and environmental factors will be essential to attaining a more comprehensive understanding of endometriosis and providing new approaches to prevent and treat the disease not only in Asians but also in other ethnic groups.

## Declaration of interest

The authors declare no conflict of interest that could be perceived as prejudicing the impartiality of the research reported.

## Funding

This paper was funded in part by the University of the Philippines Office of the Vice President for Academic Affairs (OVPAA) Emerging Interdisciplinary Research Program (EIDR-C08-006, MCV).
